# In-Situ Investigation on Nanoscopic Biomechanics of *Streptococcus mutans* at Low pH Citric Acid Environments Using an AFM Fluid Cell

**DOI:** 10.3390/ijms21249481

**Published:** 2020-12-13

**Authors:** Linh Thi Phuong Nguyen, Bernard Haochih Liu

**Affiliations:** Department of Materials Science and Engineering, National Cheng Kung University, Taiwan No.1 University Road, East District, Tainan 701, Taiwan; n58077100@gs.ncku.edu.tw

**Keywords:** *Streptococcus mutans*, atomic force microscopy, citric acid, bacterial acidic resistance mechanisms, adhesion

## Abstract

*Streptococcus mutans* (*S. mutans*) is widely regarded as the main cause of human dental caries via three main virulence factors: adhesion, acidogenicity, and aciduricity. Citric acid is one of the antibiotic agents that can inhibit the virulence capabilities of *S. mutans*. A full understanding of the acidic resistance mechanisms (ARMs) causing bacteria to thrive in citrate transport is still elusive. We propose atomic force microscopy (AFM) equipped with a fluid cell to study the *S. mutans* ARMs via surface nanomechanical properties at citric acid pH 3.3, 2.3, and 1.8. Among these treatments, at pH 1.8, the effect of the citric acid shock in cells is demonstrated through a significantly low number of high adhesion zones, and a noticeable reduction in adhesion forces. Consequently, this study paves the way to understand that *S. mutans* ARMs are associated with the variation of the number of adhesion zones on the cell surface, which is influenced by citrate and proton transport. The results are expected to be useful in developing antibiotics or drugs involving citric acid for dental plaque treatment.

## 1. Introduction

*Streptococcus mutans* (*S. mutans*) is a Gram-positive bacterium that is widely regarded as the main cause of human dental caries. These anaerobic bacteria produce acid, which is a part of the bacterial biofilm that mostly causes dental plaque or discharges virulence factors, specifically hemolysin [1,2,3,4,5,6]. *S. mutans* can also cause endocarditis, bacteremia, or heart valve isolation, and it can even lead to other systemic diseases [7]. Furthermore, the bacteria generate weak organic acids as by-products to lower the pH value in the host body or its environments [6,8]. To exhibit virulence capability, *S. mutans* utilizes three main determinants: adhesion, acidogenicity (acid production), and aciduricity (acid tolerance) [9,10]. Acidogenic *S. mutans* can produce extracellular polysaccharides (EPSs) in the presence of sucrose, fructose, and glucose [6]. The presence of EPSs in biofilm is a weapon to maintain the persistence of bacterial infection through the reduction of bacterial sensitivity to the signaling system of the host, antibiotics, and even drugs [10].

It is possible to study the mechanics of bacteria to develop impactful targets that can be used to restrict *S. mutans* infections [11]. Bacterial adhesion and the consequent development of biofilm are of major significance in biotechnology and medicine [12,13]. Elastic modulus is measured in MPa units and exhibits the rigidity of the material under an applied force [14]. The cell surface is associated with a large dispersion of the elastic moduli, since the cell membrane consists of heterogeneous composition [15]. Many diagnostic tools have been developed to investigate the mechanical force–cell structure relationship, such as optical stretchers, magnetic twisting cytometry, micropipette aspiration, and atomic force microscopy (AFM) [16,17,18,19]. Owing to the high resolution in nanoscale, and simple performance, AFM is well known as a powerful technique for imaging the surfaces of living bacteria in liquids [20,21,22]. In addition, an AFM equipped with a fluid cell can achieve accurate measurements with high specimen morphology resolutions [23,24,25]. A fluid cell is a fluid-containing chamber that immobilizes a specimen carrier during AFM operation [26]. The use of a fluid cell assists in the frequent exchange of liquid and helps with avoiding having to restart the calibration of AFM modes [23,24,26,27,28,29,30,31,32,33]. A few examples of self-designed fluid cells have been reported for biological studies, especially in living bacteria [23,24,32,33,34,35]. Notably, Kasas et al. introduced a fluid cell from polydimethylsiloxane (PDMS) using a stainless-steel mold with an elaborated syringe system to analyze cellular stiffness [23,24]. Polymers can be favorable substitutes for the traditional materials used for fluid cell production. Herein, we propose a 3D-printed fluid cell with a ring-shaped channel associated with two symmetrical pipes that can immobilize the bacteria substrate carrier with a small shear force.

Antimicrobial organic acids are good candidates for inhibiting the development of organisms. They are fully protonated species that can freely cross cell membranes as long as the external pH level is lower than the intracellular pH level of the bacteria [36]. Citric acid is an organic acid that has been used in applications such as food, cosmetics, and dietary supplements [9,37]. It is also an agent that inhibits the survival of bacteria [9]. In addition, this acid is regarded as an important intermediate in metabolism, like the Krebs cycle [38]. *S. mutans* combats the destruction by citric acid via acidic tolerance mechanisms, such as proton pumps, macromolecular reconstruction or defense, biofilm development, cell density, and regulatory systems, as well as alterations in metabolic pathways and secondary metabolism. Past studies, such as those providing evidence from a microarray analyzer, proteomics, and bioinformatics, as well as genetic expression assays, have indicated that *S. mutans* has a strong ability to survive in an acidic environment through this acid tolerance [1,9,36,38,39]. Currently, many questions remain unanswered, such as the extent of bacterial capabilities to defend against acid destruction as a concerted operational unit, and its involvement in a mixed-species community such as that represented by dental plaque [1]. Moreover, a few techniques can be used to observe the response of *S. mutans* to citric acid via the individual cell surface. Further research on the antibacterial mechanisms of citric acid on bacterial surfaces and the acidic resistance mechanisms of *S. mutans* is essential [1,36,39]. Accordingly, we utilized AFM to understand the mechanisms of citrate transport and acid resistance in *S. mutans* through characterizing the nanomechanical properties of the bacteria under citric acid at low pH levels.

In this study, we investigated the acid resistance mechanisms of *S. mutans* to low pH citric acid environments by monitoring adhesion forces in real time. We also evaluated the performance of AFM with a 3D printed fluid cell to achieve accurate measurements in liquid. The nanomechanical behaviors of *S. mutans* were characterized by using a powerful AFM operating mode called PeakForce QNM (quantitative nanomechanical property). We selected citric acid with three low pH levels of 3.3, 2.3, and 1.8 for the bacterium treatment, since *S. mutans* can survive even briefly under extremely acidic conditions (pH 2.5) [36,40]. Moreover, we referred to the initial characteristics of the bacteria in the air and phosphate-buffered saline (PBS).

## 2. Results and Discussion

### 2.1. The Prototypes of the 3D-Printed Fluid Cell

**Figure 1:**
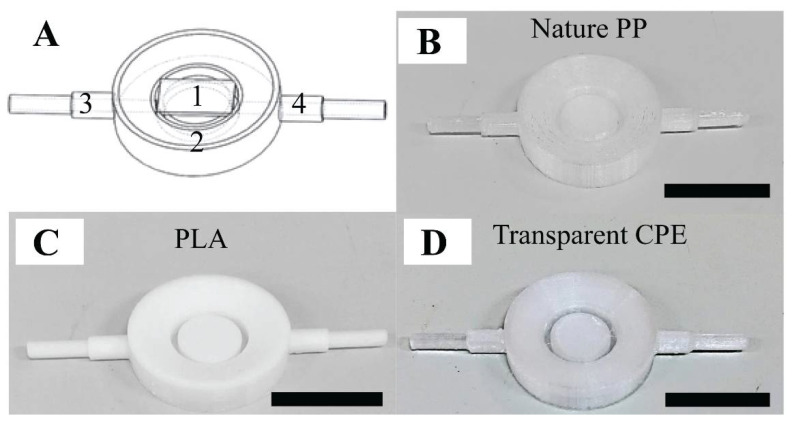
The performance of the 3D-printed fluid cell: (**A**) blueprint of the fluid cell: part (1) a glass substrate placed on top of the sample substrate holder, part (2) the ring of the fluid cell, and parts (3–4) two symmetrical pipes, and (**B**,**C**,**D**) fluid cell products involving nature polypropylene (PP), polylactic acid (PLA), and transparent copolyester (CPE), respectively. The scale bar is 1 cm.

The 3D-printed AFM fluid cell consisted of three components (see Figure 1A). (1) A glass substrate was placed on top of a sample substrate holder. (2) The fluid cell ring was the main body of the fluid cell and was around the holder. We fabricated a ring-shaped channel connected to two symmetrical pipes (3–4) via the gaps, to facilitate smooth fluid flow. The sample substrate was fitted on the holder surface, which was located at the center of the fluid cell ring and connected to these pipes. In addition, we attempted to eliminate the effect of the liquid evaporation during the AFM measurement by creating a slope in toward the center of the substrate holder from the outer-ridge surface of the ring. Accordingly, this enhanced the accuracy of the AFM measurements, since bacteria were thoroughly immersed in liquid. The liquid was injected through the pipe (3) of the fluid cell so that it would rise upward to cover the bacteria from the space between the outer ring and substrate holder. Referring to PDMS fluid cell proposed by Kasas et al., the blueprint of this 3D-printed fluid cell could immobilize the specimen under reduced shear stress [23] (see Supporting Information Sections S1 and S2). It was important that the mass flow rate be sufficiently small to be able to immobilize the specimen during the fluid exchange and scanning processes. Among the three filaments, transparent copolyester (CPE) exhibited the best performance due to its excellent chemical resistance and durability (see Figure 1B–D). Transparent CPE fluid cells were used for the purpose of examining the acid resistance mechanism of *S. mutans* suspended under liquid conditions.

### 2.2. Characterization of S. Mutans Biofilm

**Figure 2:**
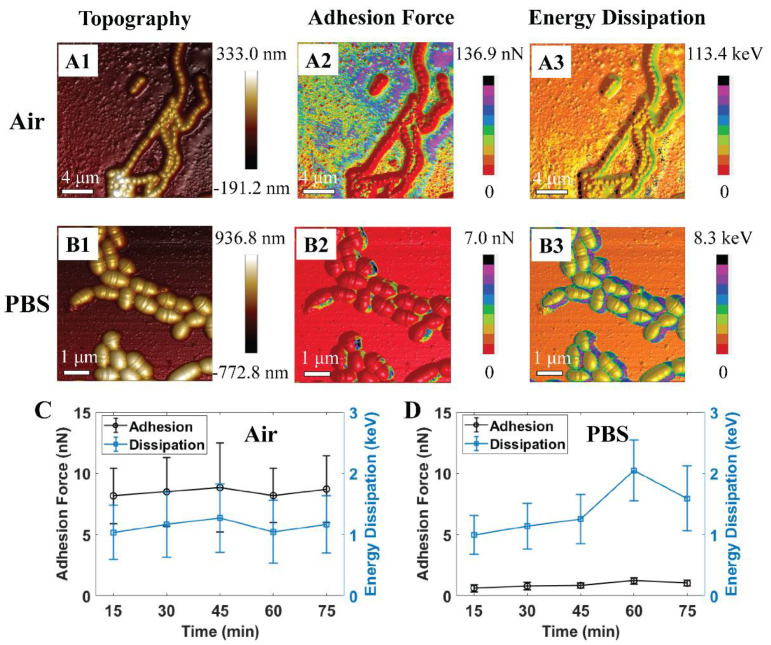
Schematic illustrations of 3D atomic force microscopy (AFM) morphologies of *S. mutans* biofilm at initial treatment in (**A**1–**A**3) air and (**B**1–**B**3) phosphate-buffered saline (PBS). Graphs of quantitative adhesion force and energy dissipation of *S. mutans* treated in (**C**) air (*n* = 57) and (**D**) PBS (*n* = 12).

Previously, we found *S. mutans* can probably survive even after one hour of exposure to air, while PBS is known to maintain bacterium growth rate and survivability [41]. This study demonstrated numerous high adhesion zones that were occupied at the cell rim and inter-cell regions (see Figure 2(A1–B3)). It was also observed that the adhesion force and energy dissipation of *S. mutans* exhibited homologous characteristics in air and PBS, respectively. Our past works using fluorescent test and AFM to investigate the nanomechanical properties of the *S. mutans* biofilm indicated that the new EPS discharge zones are associated with high adhesion emerging on the surface of bacteria, especially at the cell rim and inter-cell regions [42]. In addition, these studies indicated that an *S. mutans* biofilm contains more liquid and stickiness in the EPS zones once the high adhesion force has occurred. Therefore, the recent data suggest that nanoscopic mechanics of the rim and inter-cell zones of the *S. mutans* surface might be related to the new EPS discharge. Previously, we reported that the discharge of EPSs is one of the essential metabolic activities that these bacteria utilize to survive [42]. Adhesion forces of *S. mutans* in the air are measured higher than that of PBS, since the capillary force presented in the region of a capillary bridge forms between the tip and bacterium surface in liquid measurement [43]. These high adhesion zones are comprised of a large amount of liquid as well as stickiness. The graphs shown in Figure 2 C and D show that the adhesion force revolution was proportional to the change in energy dissipation. These investigated results are consistent with our past studies [41,42,44]. We studied the nanomechanical properties of the bacteria through their mean quantitative values to summarize the trend in evolution at various time intervals. Thus, the graphs in this manuscript have large error scales due to the variability of adhesion forces and energy dissipation in each bacterium region. Consequently, PBS solution was selected as the dynamic control environment for this study of acidic mechanism resistance of *S. mutans* under pH-controlled citric acid.

**Figure 3:**
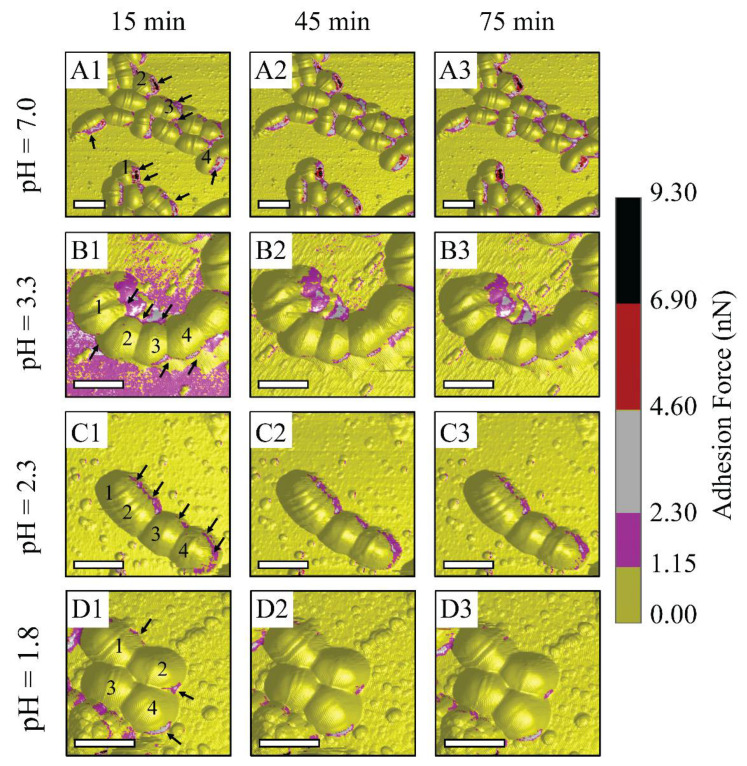
Schematic illustrations of AFM observations on *S. mutans* biofilm treated under control PBS and controlled-pH citric acid at 15 min intervals: (**A**1–**A**3) PBS, (**B**1–**B**3) citric acid pH 3.3, (**C**1–**C**3) citric acid pH 2.3, and (**D**1–**D**3) citric acid pH 1.8. (For 3D adhesion force images, scale bar is 1 μm).

Figure 3 suggests that bacterium metabolic activities might be inhibited at low pH levels of 2.3 and 1.8, which corresponds to the reduction in the number of high adhesion zones and adhesion values. The metabolic activities might be reflected through EPS discharge phenomena, since EPS is a key to maintain low bacterial sensitivity to the antibiotic [10,42]. Accordingly, a further measurement of the metabolic activities of *S. mutans* is needed to confirm our hypothesis. Under the PBS condition, there were numerous adhesion zones at bacterium inter-cell and rim areas, labeled with arrows (see Figure 3(A1–A3)). During this treatment, there were no remarkable changes in the substrate around the bacterium cells. This indicates that PBS is an environment that can maintain *S. mutans* survival. In other words, citrate and hydrogen ions dissociated from citric acid are transported and metabolized through cell membranes. At pH 3.3, numerous high adhesion zones were seen at inter-cell areas and even large areas surrounding bacterial rims, labeled with arrows (see Figure 3(B1–B3)). The number of these zones was reduced at pH level 2.3 and was vigorously lower at pH 1.8. However, numerous adhesion zones were still observed along the bacterium rim at pH 2.3 (see Figure 3(C1–C3)). Citric acid pH 1.8 tended to inhibit the metabolic activities of bacteria due to a significantly low number of narrow discharge zones (see Figure 3(D1–D3)). This was also due to the greatly reduced adhesion forces of *S. mutans* compared to those of the control PBS, and pH levels of 3.3 and 2.3. This suggests that the metabolic activities of *S. mutans* become ineffective at citric acid pH 1.8.

**Figure 4:**
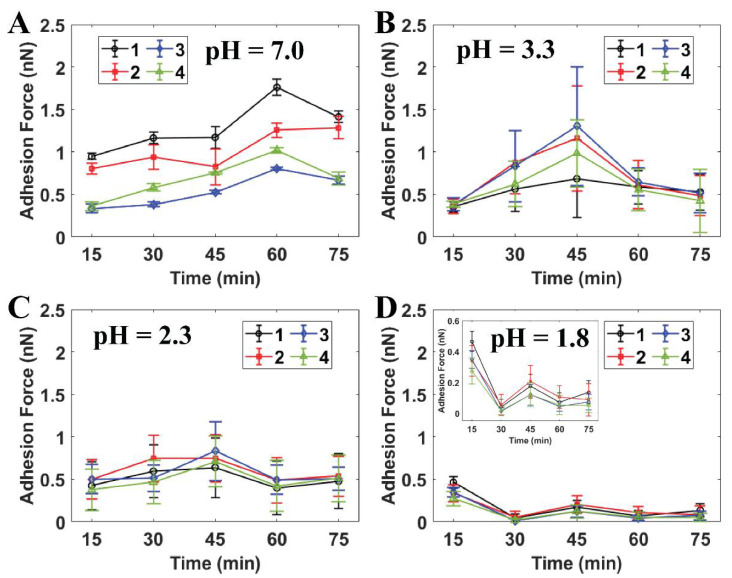
Quantitative adhesion forces of four individual *S. mutans* cells measured by AFM at (**A**) PBS pH 7.0, (**B**) citric acid pH 3.3 (*n* = 20), (**C**) citric acid pH 2.3 (*n* = 50), and (**D**) citric acid pH 1.8 (*n* = 50). Note: Cells 1–4 are labeled as individual cells in Figure 3.

Figure 4 indicates that the in situ time-lapse adhesion forces of the individual cells were reduced dramatically after the initial treatment at pH 1.8 throughout the AFM measurements (see Supporting Information Section S3). The adhesion force of *S. mutans* at this pH level was much lower than the values estimated for PBS and citric acid pH ≥ 2.3. The bacteria were therefore able to resist the strength of the citric acid solution at pH levels higher than 1.8. In particular, the adhesion forces determined under each pH level at the final stage were still maintained as well as those of the initial AFM measurement. At pH 1.8, the effect of extracellular acid shock in the cells was precisely presented, since the adhesion forces of *S. mutans* decreased throughout this experiment. Notably, these values dramatically dropped from [0.27:0.46] nN at the initial point to [0.01:0.05] nN at the 30th minute (see Figure 4D). Moreover, the initial adhesion values of *S. mutans* at pH 1.8 were the same as those at the initial measurements at pH ≥ 2.3. As a result, the citric acid shock in cells at pH 1.8 was effective in response to *S. mutans* through the significant reduction of its adhesion with respect to observation time.

**Figure 5:**
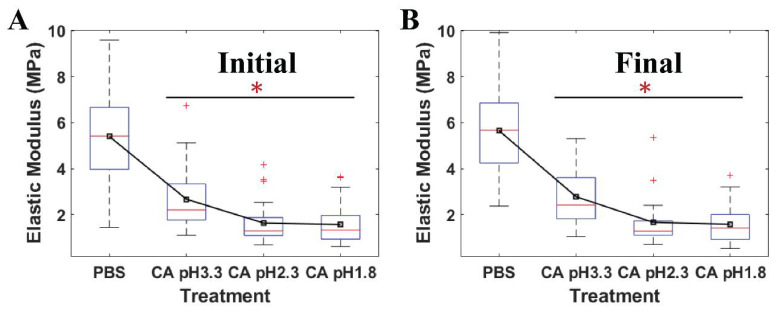
The quantitative elastic modulus of four individual *S. mutans* cells measured by AFM at (**A**) the initial stage and (**B**) the final stage under PBS pH 7 (*n* = 36), citric acid (CA) pH 3.3 (*n* = 30), citric acid 2.3 (*n* = 30), and citric acid 1.8 (*n* = 30). * Value was statistically significant among the elastic moduli of *S. mutans* treated in PBS and low pH citric acid environments, *p* < 0.001.

Figure 5 shows that the average moduli of *S. mutans* treated at low pH citric acid environments were statistically significant when compared to those of the relative control PBS. The elastic modulus mirrored the resistance of the bacteria to an applied force of the AFM probe tip. Owing to this, the ability of *S. mutans* to resist the invasion of citric acid at different pH levels was reflected in the variations of these cell moduli. Herein, a downturn in the pH levels caused a reduction in the elastic moduli emerging on the *S. mutans* surface. The moduli of the bacterial surface at the low pH citric acid were three-to-four folds lower than that of the PBS (*p* < 0.001). In addition, no significant changes in the time-lapse stiffness of *S. mutans* at the initial and final stages were investigated. Accordingly, the bacterial elastic moduli were reduced due to the presence of citric acid, suggesting that acidic stress might inhibit the bacterial ARMs more at lower pH levels. These results are consistent with the dramatic alleviation in adhesion forces of the *S. mutans* treated at low pH citric acid. Therefore, the bacterial surface treated at low pH citric acid was less sticky and was softer than that of the control PBS.

**Figure 6:**
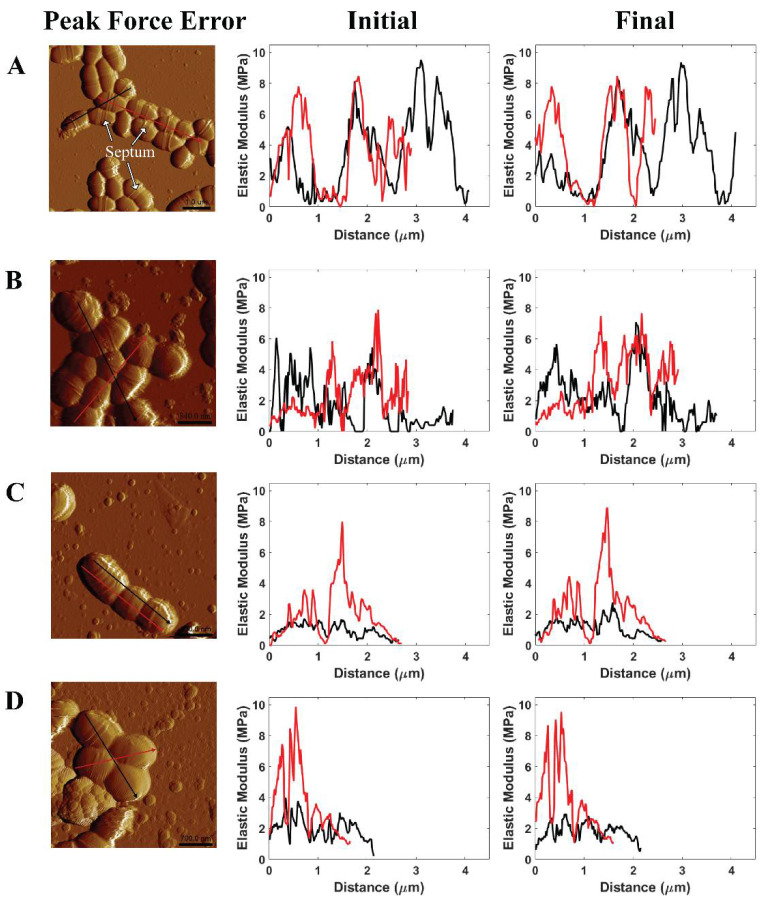
Illustrated AFM phase image and cross-sectional profile of the modulus of *S. mutans* measured in (**A**) PBS pH 7, (**B**) citric acid pH 3.3, (**C**) citric acid pH 2.3, and (**D**) citric acid pH 1.8 at the initial stage and the final stage of AFM experiments.

Generally, the events of cell division were not found at all pH level measurements due to no noticeable changes in the number of the septum (see Figure 6, peak force error). The septum, also named the Z-ring, is located in the cell where the cell division process occurs [44]. In addition, this work evaluated the effects of citric acid on *S. mutans* cells through moduli of individual cell zones at the initial and final stages of the 75 min treatment (see Figure 6). The AFM cross-sectional profiles of the elastic moduli provided an insight into the variations of the bacterial stiffness at specific regions. At pH 1.8, three among four individual cells exhibited lower elastic moduli at the final stage than the initial stage. The reduced elastic moduli of cells were measured at lower pH levels. The decrease in pH values caused a corresponding increase in the number of citrate and hydrogen ions, per unit time, passing into the cell. As a result, a reduction in pH level led to a higher potential for citric acid to attack *S. mutans* ARMs than an increase in time at each pH level due to our static treatment. There were no remarkable variations in these moduli at the initial and final stages of the treatment at pH ≥ 2.3. Consequently, the results of modulus and adhesion observations provided the same evidence of citric acid shock in cells at pH 1.8. Citrate initiates the Krebs cycle to produce nicotinamide adenine dinucleotide (NADH), hydrogen ion (H^+^), and flavin adenine dinucleotide (FADH_2_) in order to activate an electron transport chain that produces adenosine triphosphate (ATP) energy and water as a by-product [36,45]. The penetration of numerous citrate and hydrogen ions passing through the cell membrane can increase the amount of water contained in the cell. As such, the cell modulus is lower while the pH level is decreased due to the increased presence of water. At pH 1.8, the time-lapse moduli of *S. mutans* at the final stage was less than that of the initial stage, which indicated the inhibition of the bacterial acid tolerance.

### 2.3. Discussion

**Figure 7:**
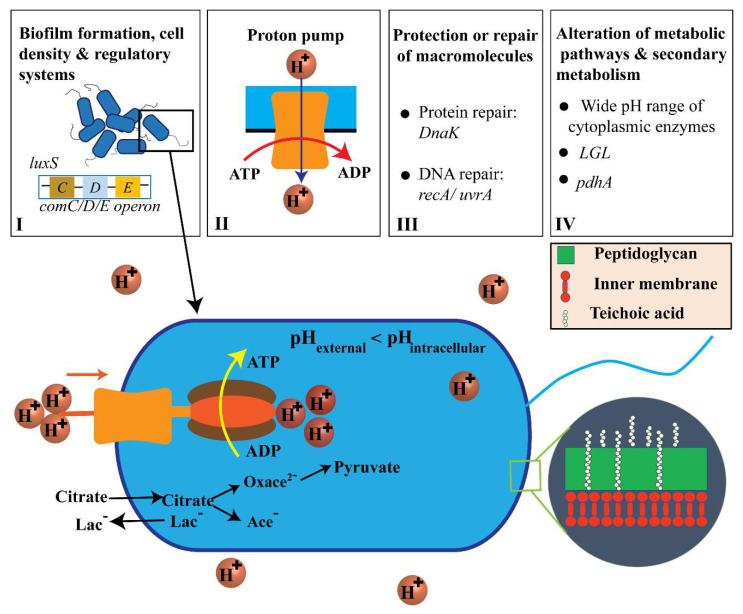
Adenosine triphosphate (ATP) generation by transport and proton-consuming decarboxylation from citric acid and the acid-resistance mechanisms (ARMs) used by *S. mutans* (not to scale). [1,2,36,46,47].

Figure 7 shows the acidic resistance mechanisms of *Streptococcus mutans* that involve (I) biofilm development, cell density, and regulatory systems; (II) proton pumping; (III) macromolecular reconstruction or defense; (IV) alterations in metabolic pathways and secondary metabolism (see Supporting Information Sections S4 and S5). Furthermore, ATP energy was generated via citrate and hydrogen ions dissociated by citric acid, oxaloacetate (Oxace^2−^), and pyruvate [1,2,36,46,47].

Biofilm formation, cell density, and regulatory systems are all important factors associated with *S. mutans* ARMs. Specifically, a biofilm can support living cells in the innermost part of the biofilm and protect cells against extracellular acid shock [48]. Cell density can be monitored through quorum sensing via *comC/D/E* operon and *luxS*, which utilize two-component regulatory systems [1,40,49]. The proton pump has been observed to be transcriptionally upregulated in *S. mutans,* which resists acidic conditions, indicating its critical role in acid resistance by hydrolyzing ATP to vigorously pump protons out of the cytosol [1,2,39]. *S. mutans* also performs glycolysis and shows ATPase-associated proton transport at pH values from 2.5 to 3.0 [36,40]. However, the external pH has not been shown to have any effect on the transcription start sites for the ATPase *operons* in *S. mutans* [36]. In acidic environments, the intracellular cytoplasm is acidified to destroy the structure of proteins or DNA molecules in terms of general function maintenance. Increasing the synthesis of protein-repair chaperone *DnaK* can result in many effects related to the reconstruction of acid-caused cellular damage in *S. mutans* [2]. For instance, it can result in higher performance of the eukaryotic signal detection particle gene *Ffh* and production of ammonia by the *Agd* system and *Adi* pathway [2] (see Supporting Information Section S6). Increases in *DnaK* also improves amino acid metabolism, alterations in metabolic pathways, induction of H^+^-ATPase, upregulation of DNA damage regulatory-repair protein *RecA*, and upregulation of alkaline phosphatase endonuclease activity [2]. Alterations in metabolism in *S. mutans* can be expressed in such a way that the bacteria metabolize various sugars into lactic acid even at low external pH values [1,9]. Secondary metabolism demonstrates that citrate serves as the entry point of citric acid into the bacterial membrane. The lactate antiporter system brings about elevated levels of acidurance by initiating a proton motive force [1,46].

Citric acid alters the local pH environment via dissociating into citrate and a hydrogen ion in order to freely pass through the cell membrane due to the higher external acidity. General bacteria neutralize intracellular pH via metabolizing various sugars into lactic acid (Lac), due to the presence of citrate and hydrolyzing ATP, to vigorously pump protons out of the cytosol [1,9,50]. However, a prior study detailed that external pH does not have any effect on the transcription start sites for the ATPase operons in *S. mutans* [36]. Additionally, the membrane of *S. mutans* can adjust the fatty acid composition as an acid-adaptive mechanism to avoid the passive inflow of H^+^ ions [51]. This changes the fatty acid composition of the cell membrane and increases end-product flow through lactic acid and proton pumping [1,36,39]. Therefore, current work aims to study the mechanisms of the metabolism pathway, biofilm formation, and macromolecular defense of *S. mutans* that correlate to its nanomechanical properties. Prior work has shown that low pH citric acid can cause microorganism death through inhibiting NADH oxidation and can lead to damage to enzymatic activities, protein, deoxyribonucleic acid (DNA), and extracellular membranes [38]. This study with AFM is limited to identifying the in situ chemical composition of the *S. mutans* membrane in nanoscale. We physically observed a great reduction in adhesion forces and a significantly low number of high adhesion zones emerging on the cell surface throughout bacteria treatment at pH 1.8. This suggests that acidic resistance of *S. mutans* at pH 1.8 is inhibited due to penetration of the citrate and hydrogen ions that pass freely into the cell membrane. In addition, the intracellular pH level of *S. mutans* is somewhat higher than that of the extracellular environment to prevent the destruction of acid-sensitive DNA and enzymes. Cotter et al. and Li et al. have identified that *S. mutans* can survive readily at pH levels of 4.5–5 and even briefly under extremely acidic conditions (pH 2.5) [36,40]. As such, it can be inferred from our findings that the bacteria still survive at low pH citric acid; however, their acidic tolerance is inhibited, especially at pH 1.8. Since an acidic environment might result in damage to the enamel of teeth, further studies are needed on metabolic activities and the dynamic treatment of low pH citric acid environments using AFM in liquid. These studies would help extend knowledge of *S. mutans* ARMs and citrate transport.

## 3. Materials and Methods

### 3.1. Fluid Cell Preparation

The blueprint of the fluid cell was constructed by SolidWorks 2018. The 3D-printed AFM fluid cells were produced from three different kinds of commercial thermoplastic filaments: polylactic acid (PLA), transparent copolyester (CPE), and natural polypropylene (PP) (3DMART LTD, New Taipei, Taiwan). An Ultimaker 3 Extended 3D printer (3DMART Ltd., New Taipei, Taiwan) with nozzles measuring 2.85 mm in diameter was employed. We mounted an adhesion sheet on the printing bed to immobilize the PP fluid cell, since PP filaments have poor adhesion. For the CPE and PLA products, the cavities of fluid cell tubes were filled with polyvinyl alcohol (PVA). This is one of the most widely used materials as a supporting part for multi-extrusion 3D printing as it is a petroleum-based, water-soluble thermoplastic. The 3D specimen was immersed completely in water to dissolve the PVA [52,53]. Multi-extrusion using PVA improves the quality of the printed fluid cells and aqueous leakage. To pre-process an AFM measurement, the fluid cell was subjected to UV light overnight to kill the organisms [34].

### 3.2. Biological Specimen Preparation

The *Streptococcus mutans* strain (ATCC 25175) was purchased from the Bioresource and Collection Research Center (Hsinchu, Taiwan). The bacterial sample was prepared according to the process described in our previous studies [42]. The *S. mutans* cells were harvested from a Bacto^TM^ brain–heart infusion (BHI) agar plate after two days and then continuously sub-cultured in a 6 mL BHI solution for approximately 16 h in an incubator at 37 °C. To process an AFM measurement, the microcolony cells were deposited onto a silanized glass substrate for 30 min as specified in our previous works [23,54]. Afterwards, we marked the substrate at the center of the fluid cell and injected acid solution for an AFM measurement.

### 3.3. AFM Characterization

The nanomechanical properties of *S. mutans* were characterized using AFM Bruker Dimension ICON AFM (Bruker Corp., Massachusetts, USA). All images were collected using a fluid cell equipped with a new PeakForce Quantitative Nanomechanical (QNM) mode. We utilized AFM tip models SCANASYST-FLUID PLUS and SCANASYST-AIR (Bruker Corp., Massachusetts, USA) for the measurements in liquid and air, respectively. The calibrated force constants of the probes were calibrated in the range of 0.10–0.61 N/m by using thermal tune function, while the tip radii of less than 80 nm were set by the relative method. The AFM probe tips and the fluid cells were dipped into 75% ethanol to remove dirt. Citric acid powder and PBS were purchased from PanReac AppliChem (Darmstadt, Germany) and Sigma-Aldrich (Missouri, USA), respectively. To eliminate the rapid displacement by the cantilever and achieve our target of investigating the local mechanics of the individual cell, the scan rate and sample scan line were adjusted to 0.3 Hz and 256, respectively. In addition, the scan size of the liquid AFM images was manipulated to be less than 10 µm. More than 150 AFM images were obtained, even though we only presented the most representative illustrations here.

The AFM data analysis was carried out using NanoScope software. Finally, we used Python to support quantitative data analysis progress. In this work, we investigated multiple nanomechanical properties of *S. mutans* involving adhesion force, energy dissipation, and elastic modulus. Specifically, an adhesion map presents the effect of environmental conditions on the bacterium surface via the tip-sample surface interactions. The quantitative adhesion is defined as the vertical distance between the baseline and the lowest position of the retraction curve in the force–distance curve used to provide absolute values [42,55]. Energy dissipation during the tip-sample interactions is the area between a trace and the retrace curves, which represents the energy difference between the engaging half-cycle and the withdrawing half-cycle [42]. The elastic modulus of the bacterium is calculated using the Derjaguin–Muller–Toropov (DMT) model of tip-sample contact using the following equations [42,56]:

(1) $$F=E_{tot}\sqrt{R}\delta^{1.5}-F_{adh}$$

(2) $$\frac{1}{E_{tot}}=\frac{3}{4}\left(\frac{1-\nu_{specimen}^{2}}{E_{specimen}}+\frac{1-\nu_{tip}^{2}}{E_{tip}}\right)$$

(3) $$a^{2}=R\delta$$

(4) $$F_{adh}=2\pi W_{adh}R$$

where F is the acting force on the tip, and $E_{tot}$ is total modulus, which is defined as the relation among elastic modulus $E_{specimen}$, Poisson’s ratios of the tip $\nu_{tip}$, and specimen $\nu_{specimen}$. Herein, the contribution of the tip is neglected in the modulus calculation. The Poisson’s ratio of *S. mutans* was selected to be 0.5, in the typical range [0.4:0.5] used in biofilm studies [57]. The DMT model is applied to calculate elastic modulus by considering the tip radius R, indentation depth δ, and adhesion force $F_{adh}$. Moreover, $W_{adh}$ and a are work of adhesion and contact radius, respectively.

### 3.4. Statistical Analysis

The statistical test, One Way Analysis of Variance (ANOVA) was performed using MATLAB, which presents the variations of the cell mechanics among the relative control PBS (pH 7.0) and citric acid at low pH levels. The significance of variances is determined through the *p*-value.

## 4. Conclusions

This work proposed AFM with a 3D-printed fluid cell to investigate the continuous in situ response of *S. mutans* in low pH citric acid environments. The high-resolution morphologies of the bacteria were recorded, since the ring-shaped fluid channel was able to immobilize the specimen carrier. At pH 1.8, the bacterial ARMs associated with a variation of the number of high adhesion zones on the cell surface were influenced by the acid shock in cells. Meanwhile, numerous high adhesion zones emerged on the cell surface at pH ≥ 2.3. The reduction in pH level resulted in a modulus decrease due to the presence of water contained in the cell. Generally, the acidic resistance of the bacteria was inhibited at pH 1.8 due to the penetration of numerous citrate and proton ions passing freely into the cell membrane.

In summary, this work provides a useful understanding of the acidic resistance mechanisms of *S. mutans* at low pH through the nanomechanical properties observed on the cell surface. As such, the results are expected to have a significant impact on the development of antibiotics or drugs involving citric acid for dental plaque treatment as well as on implant research on oral cavities.

## Supplementary Materials

The following are available online at https://www.mdpi.com/1422-0067/21/24/9481/s1.

## References

[1] Matsui R., Cvitkovitch D. (2010). Acid tolerance mechanisms utilized by Streptococcus mutans. Future Microbiol..

[2] Liu Y., Tang H., Lin Z., Xu P. (2015). Mechanisms of acid tolerance in bacteria and prospects in biotechnology and bioremediation. Biotechnol. Adv..

[3] Flemming H.-C., Neu T.R., Wozniak D.J. (2007). The EPS matrix: The “house of biofilm cells”. J. Bacteriol..

[4] Loesche W.J. (1986). Role of Streptococcus mutans in human dental decay. Microbiol. Rev..

[5] Becker M.R., Paster B.J., Leys E.J., Moeschberger M.L., Kenyon S.G., Galvin J.L., Boches S.K., Dewhirst F.E., Griffen A.L. (2002). Molecular analysis of bacterial species associated with childhood caries. J. Clin. Microbiol..

[6] Forssten S.D., Björklund M., Ouwehand A.C. (2010). Streptococcus mutans, caries and simulation models. Nutrients.

[7] Bao X., Yang J., de Soet J., Liu H., Gao X., van Loveren C., Deng D. (2017). Factors influencing the competition between streptococcus oligofermentans and streptococcus mutans in dual-species biofilms. Caries Res..

[8] Selwitz R.H., Ismail A.I., Pitts N.B. (2007). Dental caries. Lancet.

[9] Korithoski B., Krastel K., Cvitkovitch D.G. (2005). Transport and metabolism of citrate by Streptococcus mutans. J. Bacteriol..

[10] Resende A.H.M., Farias J.M., Silva D.D., Rufino R.D., Luna J.M., Stamford T.C.M., Sarubbo L.A. (2019). Application of biosurfactants and chitosan in toothpaste formulation. Colloids Surf. B Biointerfaces.

[11] Sandin J.N., Korotkova N., Grady M.E. (2020). Influence of cell wall polysaccharides on structure and mechanics of streptococcus mutans. Mechanics of Biological Systems and Materials & Micro-and Nanomechanics.

[12] Razatos A., Ong Y.-L., Sharma M.M., Georgiou G. (1998). Molecular determinants of bacterial adhesion monitored by atomic force microscopy. Proc. Natl. Acad. Sci. USA.

[13] Pissinis D.E., Benítez G.A., Schilardi P.L. (2018). Two-step biocompatible surface functionalization for two-pathway antimicrobial action against Gram-positive bacteria. Colloids Surf. B Biointerfaces.

[14] Elbourne A., Chapman J., Gelmi A., Cozzolino D., Crawford R.J., Truong V.K. (2019). Bacterial-nanostructure interactions: The role of cell elasticity and adhesion forces. J. Colloid Interface Sci..

[15] Even C., Marlière C., Ghigo J.-M., Allain J.-M., Marcellan A., Raspaud E. (2017). Recent advances in studying single bacteria and biofilm mechanics. Adv. Colloid Interface Sci..

[16] Kasas S., Longo G., Dietler G. (2013). Mechanical properties of biological specimens explored by atomic force microscopy. J. Phys. D Appl. Phys..

[17] Guck J., Schinkinger S., Lincoln B., Wottawah F., Ebert S., Romeyke M., Lenz D., Erickson H.M., Ananthakrishnan R., Mitchell D. (2005). Optical deformability as an inherent cell marker for testing malignant transformation and metastatic competence. Biophys. J..

[18] Lee L.M., Lee J.W., Chase D., Gebrezgiabhier D., Liu A.P. (2016). Development of an advanced microfluidic micropipette aspiration device for single cell mechanics studies. Biomicrofluidics.

[19] Septiadi D., Crippa F., Moore T.L., Rothen-Rutishauser B., Petri-Fink A. (2018). Nanoparticle-cell interaction: A cell mechanics perspective. Adv. Mater..

[20] Ding Y., Wang J., Xu G.-K., Wang G.-F. (2018). Are elastic moduli of biological cells depth dependent or not? Another explanation using a contact mechanics model with surface tension. Soft Matter.

[21] Dufrêne Y.F.J.M. (2014). Atomic force microscopy in microbiology: New structural and functional insights into the microbial cell surface. Am. Soc. Microbiol..

[22] Liang W., Shi H., Yang X., Wang J., Yang W., Zhang H., Liu L. (2020). Recent advances in AFM-based biological characterization and applications at multiple levels. Soft Matter.

[23] Kasas S., Radotic K., Longo G., Saha B., Alonso-Sarduy L., Dietler G., Roduit C. (2013). A universal fluid cell for the imaging of biological specimens in the atomic force microscope. Microsc. Res. Tech..

[24] Kasas S., Alonso L., Jacquet P., Adamcik J., Haeberli C., Dietler G. (2010). Microcontroller-driven fluid-injection system for atomic force microscopy. Rev. Sci. Instrum..

[25] Balashev K., Callisen T.H., Svendsen A., Bjørnholm T. (2011). Savinase action on bovine serum albumin (BSA) monolayers demonstrated with measurements at the air-water interface and liquid Atomic Force Microscopy (AFM) imaging. Colloids Surf. B Biointerfaces.

[26] Asakawa H., Katagiri Y., Fukuma T. (2013). Closed fluid cell with liquid-sealing mechanism for stable and flexible operation of liquid-environment atomic force microscopy. Jpn. J. Appl. Phys..

[27] Eaton P.W.P. (2010). Atomic Force Microscopy.

[28] Hansma P., Cleveland J., Radmacher M., Walters D., Hillner P., Bezanilla M., Fritz M., Vie D., Hansma H., Prater C. (1994). Tapping mode atomic force microscopy in liquids. Appl. Phys. Lett..

[29] Cross S.E., Kreth J., Zhu L., Qi F., Pelling A.E., Shi W., Gimzewski J.K. (2006). Atomic force microscopy study of the structure-function relationships of the biofilm-forming bacterium Streptococcus mutans. Nanotechnology.

[30] Murugesapillai D., Bouaziz S., Maher L.J., Israeloff N.E., Cameron C.E., Williams M.C. (2017). Accurate nanoscale flexibility measurement of DNA and DAN-protein complexes by atomic force microscopy in liquid. Nanoscale.

[31] Minary-Jolandan M., Yu M.-F. (2013). Nanomechanical imaging of soft samples in liquid using atomic force microscopy. J. Appl. Phys..

[32] Hillner P., Gratz A., Manne S., Hansma P. (1992). Atomic-scale imaging of calcite growth and dissolution in real time. Geology.

[33] Gebeshuber I.C., Holzer D., Goschke R., Aumayr F., Störi H. (2009). Development of an atomic force microscope closed fluid cell for tribological investigations of large samples in chemically aggressive environments. Proc. Inst. Mech. Eng. Part. J. J. Eng. Tribol..

[34] Yang C.-W., Lu Y.-H., Hwang S. (2013). Imaging surface nanobubbles at graphite-water interfaces with different atomic force microscopy modes. J. Phys. Condens. Matter.

[35] Motamedi R., Wood-Adams P.M. (2008). Influence of fluid cell design on the frequency response of AFM microcantilevers in liquid media. Sensors.

[36] Cotter P.D., Hill C. (2003). Surviving the acid test: Responses of gram-positive bacteria to low pH. Microbiol. Mol. Biol. Rev..

[37] Sabzi M., Afshari M.J., Babaahmadi M., Shafagh N. (2020). pH-dependent swelling and antibiotic release from citric acid crosslinked poly (vinyl alcohol)(PVA)/nano silver hydrogels. Colloids Surf. B Biointerfaces.

[38] Su L.-C., Xie Z., Zhang Y., Nguyen K.T., Yang J. (2014). Study on the antimicrobial properties of citrate-based biodegradable polymers. Front. Bioeng..

[39] Baker J., Faustoferri R., Quivey R. (2017). Acid-adaptive mechanisms of Streptococcus mutans—The more we know, the more we don’t. Mol. Oral Microbiol..

[40] Li Y.-H., Hanna M.N., Svensäter G., Ellen R.P., Cvitkovitch D.G. (2001). Cell density modulates acid adaptation in Streptococcus mutans: Implications for survival in biofilms. J. Bacteriol..

[41] Liu B.H., Li K.-L., Kang K.-L., Huang W.-K., Liao J.-D. (2013). In situ biosensing of the nanomechanical property and electrochemical spectroscopy of Streptococcus mutans-containing biofilms. J. Phys. D Appl. Phys..

[42] Liu B.H., Yu L.-C. (2017). In-situ, time-lapse study of extracellular polymeric substance discharge in Streptococcus mutans biofilm. Colloids Surf. B Biointerfaces.

[43] Sedin D.L., Rowlen K.L. (2000). Adhesion forces measured by atomic force microscopy in humid air. Anal. Chem..

[44] Liu B.H., Li K.-L., Huang W.-K., Liao J.-D. (2013). Nanomechanical probing of the septum and surrounding substances on Streptococcus mutans cells and biofilms. Colloids Surf. B Biointerfaces.

[45] Rice K.C., Turner M.E., O’neshia V.C., Gu T., Ahn S.-J. (2017). Modification of the Streptococcus mutans transcriptome by LrgAB and environmental stressors. Microb. Genom..

[46] Lemos J.A., Burne R.A. (2008). A model of efficiency: Stress tolerance by Streptococcus mutans. Microbiology.

[47] Kochan K., Perez-Guaita D., Pissang J., Jiang J.-H., Peleg A.Y., McNaughton D., Heraud P., Wood B.R. (2018). In vivo atomic force microscopy-infrared spectroscopy of bacteria. J. R. Soc. Interface.

[48] Willey J.M., Sherwood L.M., Woolverton C.J., Prescott L.M., Harley J.P. (2008). Prescott, Harley and Klein’s Microbiology.

[49] Matsumoto-Nakano M. (2018). Role of Streptococcus mutans surface proteins for biofilm formation. Jpn. Dent. Sci. Rev..

[50] Zhao X., Zhen Z., Wang X., Guo N. (2017). Synergy of a combination of nisin and citric acid against *Staphylococcus aureus* and *Listeria monocytogenes*. Food Addit. Contam. Part A.

[51] Brown S., Santa Maria J.P., Walker S. (2013). Wall teichoic acids of gram-positive bacteria. Annu. Rev. Microbiol..

[52] Tagami T., Fukushige K., Ogawa E., Hayashi N., Ozeki T. (2017). 3D printing factors important for the fabrication of polyvinylalcohol filament-based tablets. Biol. Pharm. Bull..

[53] Duran C., Subbian V., Giovanetti M.T., Simkins J.R., Beyette F.R. (2015). Experimental desktop 3D printing using dual extrusion and water-soluble polyvinyl alcohol. Rapid Prototyp. J..

[54] Chang A.C., Liu B.H. (2018). Identification of characteristic macromolecules of Escherichia coli genotypes by atomic force microscope Nanoscale Mechanical Mapping. Nanoscale Res. Lett..

[55] Obeid S., Guyomarc’h F., Francius G., Guillemin H., Wu X., Pezennec S., Famelart M.-H., Cauty C., Gaucheron F., Lopez C. (2019). The surface properties of milk fat globules govern their interactions with the caseins: Role of homogenization and pH probed by AFM force spectroscopy. Colloids Surf. B Biointerfaces.

[56] Liu B.H., Linh N.T.P., Chang A.C. (2020). Atomic force microscope nanoscale mechanical mapping. 21st Century Nanoscience—A Handbook.

[57] Safari A., Tukovic Z., Walter M., Casey E., Ivankovic A. (2015). Mechanical properties of a mature biofilm from a wastewater system: From microscale to macroscale level. Biofouling.

