# Supplementary material for: In-Situ Investigation on Nanoscopic Biomechanics of Streptococcus mutans at Low pH Citric Acid Environments Using an AFM Fluid Cell
Source: Int J Mol Sci. 2020 Dec 13;21(24):9481. doi: 10.3390/ijms21249481 (PMC7764216; doi:10.3390/ijms21249481)
Supplement: Supplementary file 1 [file ijms-21-09481-s001.pdf]

**Supporting Information**

# In-situ investigation on nanoscopic biomechanics of *Streptococcus mutans* at low pH citric acid environments using an AFM fluid cell


Linh Thi Phuong Nguyen and Bernard Haochih Liu*

Department of Materials Science and Engineering, National Cheng Kung University, Taiwan

No.1 University Road, East District, Tainan 701, Taiwan

*Correspondence: hcliu@mail.ncku.edu.tw

## S1. 3D printed AFM fluid cells

Figure S1 (a) presents a 3D printed AFM fluid cell consist of three components: (A) the sample substrate holder, (B) the center of the fluid cell ring, and (C) two pipes. The sample substrate was placed at the center of the fluid cell ring and connected to these pipes. We fabricated a ring-shaped channel connected to two symmetrical pipes via the gaps to facilitate smooth fluid flow. It is important that the mass flow rate be sufficiently small to be able to immobilize the specimen during the fluid exchange and scanning processes. A finite-element simulation (Siemens NX) was used to assess the effectiveness of fluid exchange between citric acid and water in the core of the fluid cell (See Fig. S1 b). The entire fluid cell core was initially stored in water without air, and citric acid was injected to replace the water with a velocity of 1.2 mm/s. We measured the fluidic velocity by using a syringe pump system which allows either a single injection or aspiration process. The simulated velocity of citric acid was minimized to about 0 m/s at the vicinity of the sample substrate holder. Therefore, it can be said that the fluid cell could immobilize the specimen under reduced shear stress.[1]. The detailed printing parameters were shown in Table S1.

Table S1. Parameters of the filaments used for fluid cell printing.

| Parameters | PLA | Transparent CPE | Nature PP | PVA (support) |
| --- | --- | --- | --- | --- |
| Printing temperature (°C) | 200 | 240 | 205 | 215 |
| Plate temperature (°C) | 60 | 80 | 75 | 70 |
| Fill density (%) | 40 | 50 | 40 | 20-50 |
| Chemical resistance | Fair | Excellent | Good | Dissolved in liquid |
| Support | PVA | PVA | Adhesion sheets | --- |

## S2. AFM conducted with a 3D printed fluid cell

## S3. Statistical Analysis

Table 1. Statistically significant values present the variations of the bacterial adhesion forces amongst a control PBS and each low pH citric acid. (The value 1 shows the significant difference between the adhesion of the cell treated in the control and each pH citric acid ($p < 0.05$), and value 0 presents no significant difference).

| Time (min) | CA pH 3.3 | | | | CA pH 2.3 | | | | CA pH 1.8 | | | |
|---|---|---|---|---|---|---|---|---|---|---|---|---|
| | Cell 1 | Cell 2 | Cell 3 | Cell 4 | Cell 1 | Cell 2 | Cell 3 | Cell 4 | Cell 1 | Cell 2 | Cell 3 | Cell 4 |
| 15 | 1 | 0 | 1 | 1 | 1 | 0 | 0 | 1 | 0 | 1 | 1 | 1 |
| 30 | 0 | 0 | 0 | 0 | 0 | 0 | 0 | 1 | 1 | 1 | 1 | 1 |
| 45 | 0 | 0 | 1 | 0 | 0 | 0 | 0 | 0 | 1 | 1 | 1 | 1 |
| 60 | 1 | 1 | 1 | 1 | 1 | 1 | 1 | 1 | 1 | 1 | 1 | 1 |
| 75 | 1 | 1 | 1 | 1 | 1 | 1 | 1 | 1 | 1 | 1 | 1 | 1 |

## S4. Acid resistance mechanisms in general bacteria and *S. mutans*

### General bacteria ARMs

General mechanisms of bacterial resistance to acid attack are as follows: proton pumping by $F_1$–$F_0$–ATPase, the glutamate decarboxylase system and electrogenic transport, macromolecular reconstruction or defense, biofilm development, cell density and regulatory systems; including sigma factor and two-component signal transduction, alkali production, production of deiminase and arginine deiminase and alteration of metabolic pathways and secondary ATPase activity, acid end-product flow, and lessened proton permeability–all of which allows a state of low intracellular concentration of protons.[2] Also, macromolecular repair and protection is performed via the protective function of the following genes: *recA*, *uvrA*, and *DnaK;* bacteria living in acidic conditions, comprise the surroundings necessary to acidify the intracellular cytoplasm.[2-5] Biofilms are omnipresent in nature and perform cell attachment to the host that releases polysaccharides, protein, and DNA. Under acidic conditions, the biofilm can support living cells by protecting them against extracellular acid shock. Furthermore, cell density can be monitored through quorum sensing, which harnesses two-component regulatory systems.[3,6] The bacteria can therefore generate alkali products to counterbalance the acid generated during extracellular metabolism.[2] They can also metabolize various sugars into lactic acid even at lower external pH values.[7] Changes in the cell membrane are demonstrated through alterations in the fatty acid composition.[8]

***Streptococcus mutans* ARMs**

**(1) $F_1$-$F_0$-ATPase proton pump**

*S. mutans* displaces protons to maintain a relatively neutral intercellular pH level. $F_1$–$F_0$–ATPase has been observed to be transcriptionally upregulated in *S. mutans,* which resists acidic conditions, indicating its critical role in acid resistance by hydrolyzing ATP to vigorously pump protons out of the cytosol.[2,3,9] However, the external pH has not been shown to have any effect on the transcription start sites for the ATPase operons in S. mutans.[4]

**(2) Macromolecule repair or protection**

In acidic environments, it is possible to acidify the intracellular cytoplasm in order to destroy the structure of proteins or DNA molecules in terms of general function maintenance. Increasing the synthesis of protein-repair chaperone *DnaK* can result in many effects that could possibly be related to the reconstruction of acid-caused cellular damage in *S. mutans*.[2] For instance, it can result in higher performance of the eukaryotic signal detection particle gene *Ffh* and production of ammonia by the *Agd* system and *Adi* pathway.[2] Increases in *DnaK* also improves amino acid metabolism, alterations in metabolic pathways, induction of $H^+$-ATPase, upregulation of DNA damage regulatory-repair protein *RecA*, and upregulation of alkaline phosphatase endonuclease activity.[2]

***recA*** repairs DNA and activates the emergency response.[2,3]

***uvrA*** restores damaged DNA during nucleotide-ablation reparation.[2,3]

***DnaK*** is a highly sensitive stress sensor that manages the character of heat shock genes immediately in terms of protein misfolding responsibility.[2,3]

**(3) Bacterial formation, cell density and regulatory systems**

Biofilm development, cell density, and regulator systems are all important factors associated with *S. mutans* ARM. Specifically, a biofilm can support living cells in the innermost part of the biofilm and protect cells against extracellular acid shock.[6] Cell density can be monitored

through quorum sensing via *comC/D/E* operon and *luxS*, which utilize two-component regulatory systems.[3,8,10]

Biofilms are omnipresent in nature to perform cell attachment to the host, which releases polysaccharides, protein, and DNA. These polymers allow the microbes to adhere to the surface. Biofilm can support living cells by protecting them against extracellular acid shock,.[6] Additionally, cell density can be monitored through quorum sensing, which utilizes two-component regulatory systems. In simple words, the acid resistance of biofilm cells is less significant at lower cell densities. In *S. mutans*, the cell density can be observed through the *comC/D/E* operon and *luxS* gene.[3]

The ***ComC/D/E* operon** encodes a quorum-sensing system essential for cell biofilm formation. Specifically, the *comC* operon encodes a competence-stimulating peptide (CSP) while *comD* and *comE* encode a histidine kinase and response regulator, respectively. The histidine kinase and regulator consist of a two-component signal transduction system that can detect the external CSP.[2-5,8,10]

***luxS*** mediates quorum sensing regulated biofilm formation and stress tolerance.[2-5]

**(4) Alterations in metabolic pathways and secondary metabolism**

Alterations in metabolism in *S. mutans* can be expressed in such a way that the bacteria metabolize various sugars into lactic acid even at low external pH values.[3,7] Secondary metabolism demonstrates that citrate serves as the entry point of citric acid into the bacterial membrane. The lactate antiporter system brings about elevated levels of aciduance by initiating a proton motive force.[3,11]

**Alterations in metabolism**: *S. mutans* metabolizes various sugars into lactic acid even at low external pH values.[3,7] After citric acid treatment, *S. mutans* coverts internalized citrate into the amino acid aspartate.[7] Specifically, the bacteria tend to lessen their internal pH necessary for glucose uptake by changing its metabolic activity even under harsh acidic and survival conditions.[3]

**Lactoylglutathione lyase (LGL)** is one of the many upregulated genes in *S. mutans* having a pH lower than 5.0, which indicates that it participates in acid tolerance. Furthermore, LGL is very important for

cell survival because of its detoxification of methylglyoxal, a glycolytic by-product that causes cytoplasmic macromolecule inactivity such as that in proteins and nucleic acids.[3]

***pdhA*** encodes pyruvate dehydrogenase due to acid stress and acid adaptation. If *pdhA* is lost via mutagenesis, it exhibits an acid-sensitive phenotype, and the expression of this protein closely mirrors metabolic activity, which is in line with heterofermentative growth (i.e., decreased activity when glucose is in excess).[3]

## S5. Alterations in membrane composition: membrane components necessary to maintain a physiological composition

***Ffh*** is the 54-kDa sub-unit homologue of the eukaryotic signal recognition particle.[12] Transcription of *ffh* in *S. mutans* is amplified due to acid-induced stress.[13] In *S. mutans*, *ffh* can be found inside the secretion and acid tolerance (sat) operon.[12,13]

***fabM*** is the sole gene responsible for the production of monounsaturated fatty acids in *S. mutans* Deletion of this gene results in a mutant that exhibits distinct acid-sensitive attributes when compared with the wild-type strain.[12]

***Dcp (D-alanyl carrier protein)*** in D-alanyl-lipoteichoic acid fabrication is a major macro amphiphile playing an important role in the growth and development of gram-positive organisms.[3,14]

***Dgk* (diacylglycerol kinase)** 137 amino acids homologue of *S. mutans* important for the response of the organism to acid stress.[12]

## S6. Production of Alkali

**Agmatine deiminase system (Agd system)** produces ammonia at low pH values and is regulated by agmatine induction and carbohydrate catabolite repression.[15]

**Arginine deiminase pathway (Adi pathway)** is the most commonly used anaerobic route for arginine degradation as the main source of energy used by many microorganisms, which undergoes three consecutive reactions catalyzed by arginine deiminase, ornithine transcarboxylase, and carbamate

kinase. As such, the Adi pathway performs the conversion of arginine to ornithine, ammonia, and $CO_2$, producing one mol of ATP for each mol of arginine consumed.[16]

## References


1. Kasas, S.; Radotic, K.; Longo, G.; Saha, B.; Alonso-Sarduy, L.; Dietler, G.; Roduit, C. A universal fluid cell for the imaging of biological specimens in the atomic force microscope. *Microscopy research technique* **2013**, *76*, 357-363.
2. Liu, Y.; Tang, H.; Lin, Z.; Xu, P. Mechanisms of acid tolerance in bacteria and prospects in biotechnology and bioremediation. *Biotechnology advances* **2015**, *33*, 1484-1492.
3. Matsui, R.; Cvitkovitch, D. Acid tolerance mechanisms utilized by Streptococcus mutans. *Future microbiology* **2010**, *5*, 403-417.
4. Cotter, P.D.; Hill, C. Surviving the acid test: responses of gram-positive bacteria to low pH. *Microbiology molecular biology reviews* **2003**, *67*, 429-453.
5. Kochan, K.; Perez-Guaita, D.; Pissang, J.; Jiang, J.-H.; Peleg, A.Y.; McNaughton, D.; Heraud, P.; Wood, B.R. In vivo atomic force microscopy–infrared spectroscopy of bacteria. *Journal of The Royal Society Interface* **2018**, *15*, 20180115.
6. Joanne M. Willey, L.M.S.a.C.J.W. *Prescott, Harley, and Klein's Microbiology*, Seventh ed.; 2008; pp. 55-56, 143-144.
7. Korithoski, B.; Krastel, K.; Cvitkovitch, D.G. Transport and metabolism of citrate by Streptococcus mutans. *Journal of bacteriology* **2005**, *187*, 4451-4456.
8. Li, Y.-H.; Hanna, M.N.; Svensäter, G.; Ellen, R.P.; Cvitkovitch, D.G. Cell density modulates acid adaptation in Streptococcus mutans: implications for survival in biofilms. *Journal of bacteriology* **2001**, *183*, 6875-6884.
9. Baker, J.; Faustoferri, R.; Quivey Jr, R. Acid-adaptive mechanisms of Streptococcus mutans–the more we know, the more we don't. *Molecular oral microbiology* **2017**, *32*, 107-117.

10. Matsumoto-Nakano, M. Role of Streptococcus mutans surface proteins for biofilm formation. *Japanese Dental Science Review* **2018**, *54*, 22-29.
11. Lemos, J.A.; Burne, R.A. A model of efficiency: stress tolerance by Streptococcus mutans. *Microbiology* **2008**, *154*, 3247.
12. Shibata, Y.; van der Ploeg, J.R.; Kozuki, T.; Shirai, Y.; Saito, N.; Kawada-Matsuo, M.; Takeshita, T.; Yamashita, Y. Kinase activity of the dgk gene product is involved in the virulence of Streptococcus mutans. *Microbiology* **2009**, *155*, 557-565.
13. Kremer, B.H.; van der Kraan, M.; Crowley, P.J.; Hamilton, I.R.; Brady, L.J.; Bleiweis, A.S. Characterization of the sat operon in Streptococcus mutans: evidence for a role of Ffh in acid tolerance. *Journal of bacteriology* **2001**, *183*, 2543-2552.
14. Heaton, M.P.; Neuhaus, F.C. Role of the D-alanyl carrier protein in the biosynthesis of D-alanyl-lipoteichoic acid. *Journal of Bacteriology* **1994**, *176*, 681-690.
15. Griswold, A.R.; Chen, Y.-Y.M.; Burne, R.A. Analysis of an agmatine deiminase gene cluster in Streptococcus mutans UA159. *Journal of bacteriology* **2004**, *186*, 1902-1904.
16. Zúñiga, M.; Pérez, G.; González-Candelas, F. Evolution of arginine deiminase (ADI) pathway genes. *Molecular phylogenetics evolution* **2002**, *25*, 429-444.
